# Influence of Corrosion on the Bond–Slip Behaviour between Corroded Bars and Concrete

**DOI:** 10.3390/ma16237366

**Published:** 2023-11-27

**Authors:** Chenxu Zhao, Zongquan Ying, Chengbin Du, Shuai Yang, Hansheng Liu

**Affiliations:** 1 College of Mechanics and Materials, Hohai University, Nanjing 210098, China; 2 CCCC Fourth Harbour Engineering Institute Co., Ltd., Guangzhou 510230, China; 3 Hydraulic Structure Durability Technology Key Laboratory of Transportation Industry, Guangzhou 510230, China; 4 Guangdong Provincial Laboratory of Southern Ocean Science and Engineering (Zhuhai), Zhuhai 519082, China

**Keywords:** reinforced concrete, longitudinal bar corrosion, stirrup corrosion, concrete strength, bond–slip, constitutive model

## Abstract

Pull-out tests were conducted to investigate the effects of corrosion of both the longitudinal bars and stirrups on the bond slip behaviour of reinforced concrete specimens. The main experimental variables include concrete strength (26.7 MPa, 37.7 MPa and 45.2 MPa) and expected corrosion loss (0%, 4%, 8% and 12%), with a total of 63 specimens fabricated. The results show that the relative bonding strength of specimens under different concrete strengths gradually decreases with increasing corrosion loss, but the higher the concrete strength is, the faster its degradation rate. The influence of stirrup corrosion on the peak slip can be ignored, but it will further aggravate the degradation of the bonding strength of the specimens. This reduction in bonding strength is linearly related to the stirrup corrosion loss. Based on the experimental results of this work and the achievements of other scholars, a modified relative bonding strength degradation model and a bond–slipbond–slip constitutive model of corroded reinforced concrete are presented by accounting for the influence coefficient of concrete strength. The results show that the constitutive model is in good agreement with the relevant experimental results.

## 1. Introduction

Reinforced concrete structures are widely used in modern engineering structures because of their high strength, excellent modulability, fire resistance and high-cost performance. Good bonding performance is necessary for steel bars and concrete to work together effectively. The corrosion of steel bars is an important reason for the loss of interface bonding capacity. Many scholars have conducted experimental studies on the bond slip behaviour of corroded reinforced concrete. On one hand, some studies have focused on exploring the bond slip behavior of new types of reinforced concrete, such as fiber-reinforced polymer composites, which can significantly extend the service life of concrete structures [1]. Zhou et al. [2] believe that the ability of specimens with carbon fiber reinforced polymer (CFRP) constraints to resist the degradation of bond strength caused by steel corrosion is higher than that of ordinary specimens. Kazemi et al. [3] found that under the same corrosion loss, the bonding strength of glass fiber-reinforced polymer (GFRP) specimens is higher than that of ordinary reinforced concrete specimens and the bonding strength of seawater concrete specimens decreases more severely than that of ordinary specimens. Based on the pull-out test, Ge et al. [4] obtained a bond slip model between a steel-FRP composite bar (SFCB) and concrete that considers chloride corrosion and load. Wang et al. [5] found that during the pull-out test, the concrete cover of the reinforced concrete sample using seawater and sea sand split more severely, and the confinement effectiveness of FRP-reinforced specimens is higher than that of normal specimens.

On the other hand, some studies have focused on considering the change in bond slip behaviour under the coupling of various influencing factors. In early studies, the factors considered in models were relatively simple. Zhang et al. [6], based on the pull-out test without stirrups, obtained a formula for calculating the relative bonding strength considering only the corrosion of longitudinal reinforcement and believed that the relative bonding strength decreased linearly with the corrosion loss. Fu et al. [7] found that the bond performance of specimens with the non-uniform corrosion of steel rebar degraded more significantly. Yalciner et al. [8] showed that the degradation of the interfacial bonding strength between concrete and rebar with different concrete strength grades was different and that concrete with a higher strength was more sensitive to the deterioration of bonding properties caused by corrosion. Yin et al. [9] proposed an exponential bonding strength degradation model considering the corrosion of the longitudinal bar by conducting pull-out tests on specimens without stirrups. The above models did not consider the influence of stirrups, but Feng et al. [10] noted that the existence of stirrups could significantly improve the bonding strength of a specimen and thus change the failure mode of a specimen. Zhou et al. [11] believe that a small degree of stirrup corrosion is beneficial to bonding strength, and the influence of stirrup corrosion on bonding performance is different in concrete with different strength grades. Based on the pull-out test, Lin et al. [12] proposed a bonding strength degradation model that accounted for multiple coupling factors, such as longitudinal reinforcement corrosion, stirrup corrosion and stirrup spacing, but it did not account for the influence of concrete strength, resulting in deviations in the prediction results.

Most of the previous studies were based on the corrosion of only the longitudinal reinforcement and rarely considered the effect of concrete strength on the bond–slip behaviour. However, in actual engineering, the longitudinal reinforcement and the stirrup corrode at the same time. Due to the thin concrete cover of the stirrup, the degree of corrosion of the stirrup is often more serious than that of the longitudinal reinforcement [13]. In view of this, to explore the influence of longitudinal bar and stirrup corrosion on bonding strength under different concrete strength levels, three eccentric pull-out specimens with different concrete strengths were poured in this paper and subjected to electrochemically accelerated corrosion to obtain specimens with different steel mass loss rates. Based on the pull-out test results, a modified relative bonding strength degradation model and a bond–slip constitutive model of corroded reinforced concrete were proposed, and the model results were compared with the relevant test results.

## 2. Overview of the Test

### 2.1. Experimental Design

**Figure 1:**
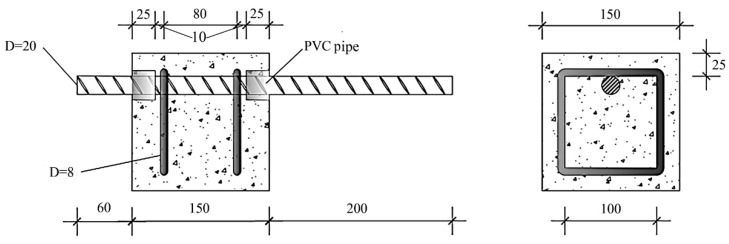
Dimensions of the pull-out test specimens (unit: mm).

The variables of this test are concrete strength grade (C25, C35 and C45), corrosion loss (0%, 4%, 8% and 12%), and whether the stirrup is rusted, with a total of 21 groups of different working conditions. To obtain more accurate results, 3 specimens were prepared for each working condition, and a total of 63 specimens were poured. The parameters of the specific specimens are shown in Table 1. The specimen size was 150 mm × 150 mm × 150 mm. Each longitudinal reinforcement was an HRB400 steel bar with a diameter of 20 mm, each stirrup was an HPB300 steel bar with a diameter of 8 mm, and the stirrup spacing was 80 mm. To obtain a reasonable bonding stress distribution, the bonding length of the longitudinal bar was 5 times the diameter of the steel bar, and the unbonded area was covered with polyvinyl chloride (PVC) pipe to prevent the generation of a high bonding stress caused by the extrusion of the test frame during the test [14], as shown in Figure 1.

### 2.2. Preparation of Test Piece

The three strength grades of C25, C35 and C45 were prepared for testing. The cement was ordinary Portland cement, the fine aggregate was river sand with a maximum particle size of 3 mm, the coarse aggregate was gravel with a maximum particle size of 20 mm, and the water was tap water. The mix ratios of C25, C35 and C45 concrete were cement:sand:gravel:water = 1:1.68:3.93:0.52, 1:1.26:2.94:0.41 and 1:1.46:3.41:0.45, respectively. After the concrete was prepared, the specimens were poured and cured for 28 days; then, cube compressive strength testing was carried out. According to GB/T 50081, the cube compressive strengths of the three kinds of concrete were 26.7 MPa, 37.7 MPa and 45.2 MPa, respectively.

**Figure 2:**
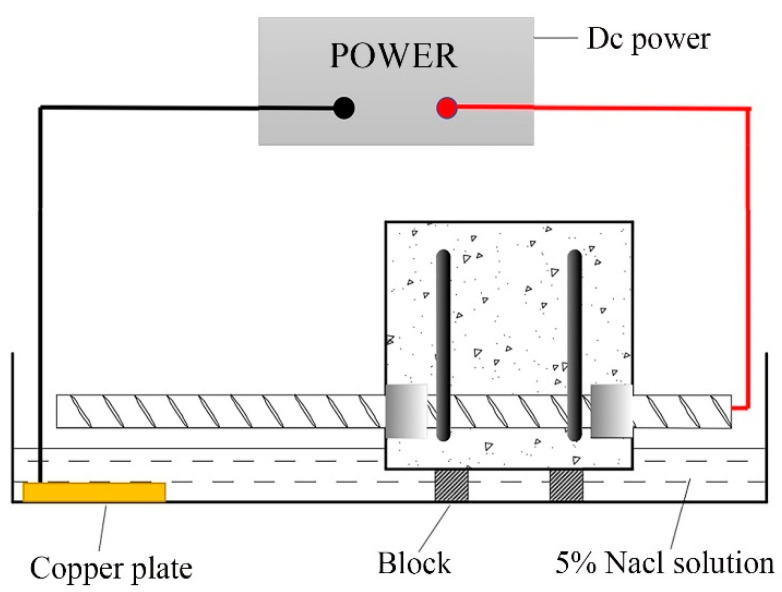
Electrochemically accelerated corrosion test.

Tian et al. [15] noted that the structural bearing capacity under the half soaking method (HSM) is basically the same as that in the natural environment. Considering the large number of specimens and the convenience of management, the HSM was chosen for the current test. Before corrosion, to supply power, the specimen was first immersed in NaCl solution with a mass fraction of 5% for 3 days, the positive electrode was connected to the free end of the specimen, and the cathode was connected to the copper sheet in water to form a loop. To ensure the liquid level height and full contact between the surface of the specimen and the solution, a pad was placed at the bottom of the specimen, and the liquid level was located 10 mm below the steel bar, as shown in Figure 2. The specimens were connected in parallel with copper wires. The current density was 0.4 mA/cm^2^. The power supply was adjusted daily to ensure the stability of the current.

The principle of Faraday’s law is that the amount of charge transferred in the electrochemical reaction is equal to the number of electrons transferred in the corroded steel bar, so the charging time of the specimen was calculated according to Faraday’s law:

(1) $$\begin{array}{c} t=\frac{∆mZF}{MI}\; \end{array}$$

where, t is the power at time (s), ∆m is the corrosion mass (g), and M is the relative molecular mass of iron, which was 56 g/mol here. I is the average current (A) used in the electrical acceleration process, and Z is the number of electrons lost by the iron atom, which was 2 here. F is Faraday’s constant, which was $9.65\times 10^{4}\;C/mol$ here.

The current needed to initiate the corrosion of each specimen was calculated to be 0.025 A, and the electrification times needed to reach the expected corrosion loss of 4%, 8% and 12% were 15.7 d, 31.5 d and 47.2 d, respectively.

The corrosion loss of the longitudinal reinforcement can be calculated using Formula (2):

(2) $$\begin{array}{c} \eta =\frac{m_{0}-m_{1}}{m_{0}}\; \end{array}$$

where, *η* is the corrosion loss of the longitudinal reinforcement, $m_{0}$ is the mass of the steel bar without corrosion in the anchoring section (g) and *m*_1_ is the mass of the steel bar after rust removal in the anchoring section (g).

The corrosion loss of the stirrup can be calculated using Formula (3):

(3) $$\begin{array}{c} \eta_{st}=\frac{m_{st0}-m_{st1}}{km_{st0}}\; \end{array}$$

where, $\eta_{st}$ is the corrosion loss of the stirrup,$m_{st0}$ is the mass of the uncorroded reinforcement in the anchoring section (g) and $m_{st1}$ is the mass of the reinforcement after rust removal in the anchoring section (g). Due to the HSM (half soaking method) used in the corrosion method, a portion of the stirrup on the side away from the solution was not corroded. Therefore, k is used to calculate the actual mass of the corrosion area, k is a dimensionless coefficient equal to the ratio of the length $l_{corr}$ of the rusted region of the stirrup to the original length $l_{0}$, that is, $k=l_{corr}/l_{0}$. $l_{0}$ is the original length of the stirrup, which was 460 mm in this experiment, and $l_{corr}$ is the length of the corroded part measured in the experiment.

### 2.3. Loading of the Specimens

**Figure 3:**
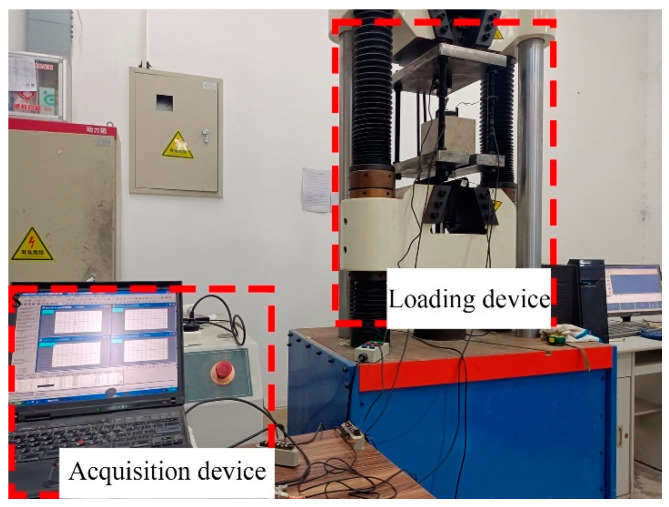
The pull-out testing set up.

The pull-out testing was completed with an electrohydraulic servo universal testing machine with a measuring range of 1000 kN, and a force sensor with a measuring range of 400 kN was used to measure the pull-out force. Two displacement sensors with a measuring range of 25 mm were set at the loading end and the free end of the concrete. The loading rate of the test was 0.5 mm/min, and the end was marked by the stability of the pull-out force or the failure of the specimen. The test site is shown in Figure 3. The bonding strength of the specimen is calculated as follows:

(4) $$\begin{array}{c} \tau =\frac{F}{\pi Dl}\; \end{array}$$

where τ is the average bonding strength, F is the pull-out force, D is the diameter of the longitudinal bar and l is the length of the bonding zone.

The slip value is expressed as the average of the displacement data of the free end and the loaded end:

(5) $$\begin{array}{c} s=\frac{s_{1}+s_{2}}{2}\; \end{array}$$

where *s* is the average slip value of the specimen, $s_{1}$ is the displacement of the free end and $s_{2}$ is the displacement of the loading end.

## 3. Results and Discussion

### 3.1. Analysis of Corrosion Test Results

**Figure 4:**
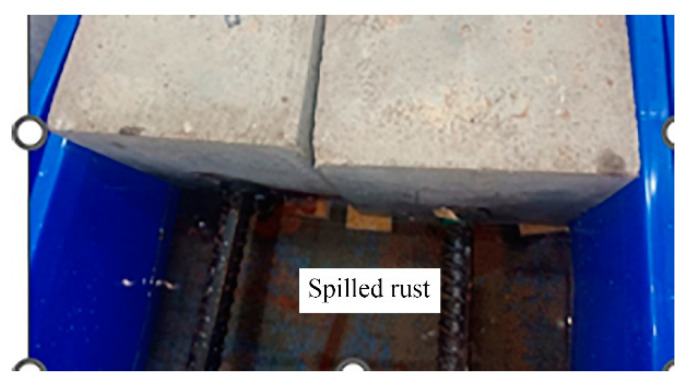
Rust overflow phenomenon.

**Figure 5:**
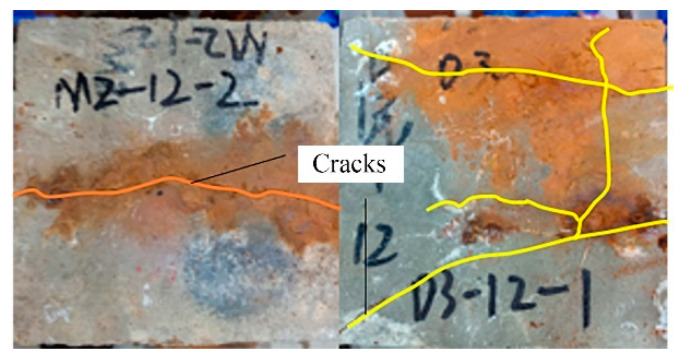
Rust expansion crack.

**Figure 6:**
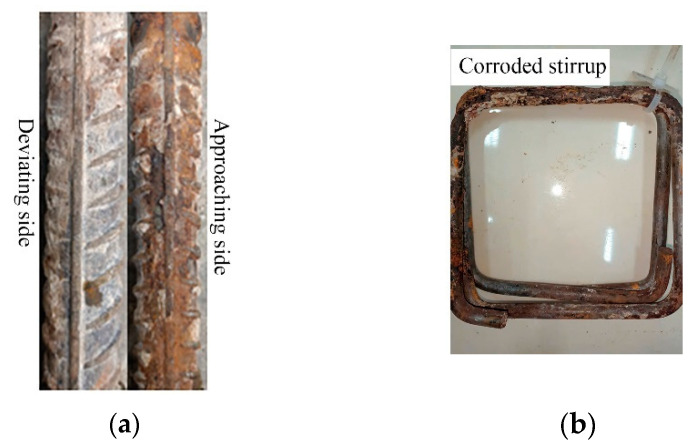
Corroded steel bars in specimens. (**a**) Corroded longitudinal bar. (**b**) Corroded stirrup.

**Figure 7:**
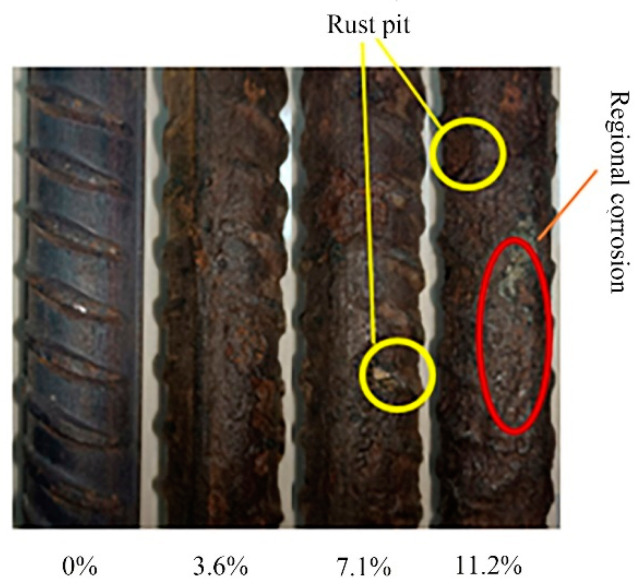
Shape of steel bars after rust removal.

During the process of energized corrosion (rusting), most of the samples showed rust overflow, as shown in Figure 4. Under the same preset corrosion loss, the D series rust overflow phenomenon was more serious than that of the M series because the D series stirrup was not insulated, the concrete cover of the stirrup was thinner, and the rust was more likely to overflow the surface and enter the solution. After the corrosion was completed, all specimens showed rust expansion cracks, and the cracking of typical specimens is shown in Figure 5. The rust expansion cracks of the M-series specimens mostly formed along the longitudinal reinforcement, while the rust expansion cracks of the D-series specimens were more random, showing that the longitudinal and transverse cracks crossed each other at the concrete surface. Because a part of the current was removed from the stirrup in the D series, which played a certain role in protecting the longitudinal reinforcement of the specimen from corrosion, the longitudinal seam on the surface of the D-series specimens was mostly smaller than that of the M-series specimens with the same expected corrosion loss. After loading, the specimens were broken, and the rebars were extracted. Figure 6a shows that the side near the concrete cover was more seriously corroded because the ion concentration on this side was higher as a result of the HSM. Figure 6b shows the stirrup with severe corrosion, and it can be seen that the cross-section loss in some areas of the stirrup was very serious. Therefore, the influence of stirrup corrosion cannot be ignored in relevant studies. The removed rebar was treated with rust removal. As shown in Figure 7, the regional corrosion and pitting of the steel bars were visible to the naked eye as the corrosion loss increased.

### 3.2. The Influence of Longitudinal Bar Rust on the Ultimate Bonding Strength

**Figure 8:**
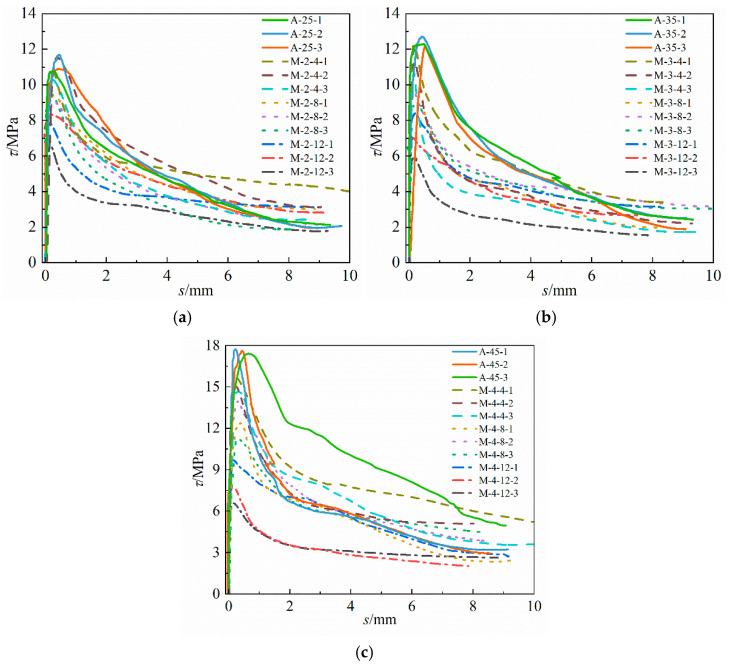
Bond–slip curves of the A-series and M-series specimens. (**a**) A-25 and M-2, (**b**) A-35 and M-3 and (**c**) A-45 and M-4.

The pull-out test results of the A-series and M-series specimens are summarized in Table 2 and Figure 8. The actual corrosion loss of the rebar was mostly lower than the expected corrosion loss, which may be caused by the loss of current in the conduction process. With the increase in the longitudinal reinforcement corrosion loss, the ultimate bonding strength of the corroded concrete specimens with different concrete strengths also decreased and only reached 40% of the uncorroded specimens in severe cases. On the one hand, the corrosion of steel bars will lead to cracking of the concrete cover. The greater the degree of corrosion is, the wider the crack width will be, which reduces the restraint effect of concrete on rebar. On the other hand, corrosion of the rebar will weaken the rib height, and the resulting rust will also play a “lubricating” role at the junction of the steel bar and concrete, thereby reducing the bonding strength. The bond–slip curve of the specimen was divided into three parts, the “rising section” (from the start of the test to the peak slip), “falling section” (from the peak slip to the slip point of 8 mm) and the “residual section” (from the slip point of 8 mm to the end), reflecting the sharp rise in the bonding stress of the specimen after the start of the test and then the relatively gentle decline to the residual stress. In addition, with the increasing concrete strength, the span of the peak points of the bond–slip curve along the abscissa also increased correspondingly. The maximum limit bonding strength of the uncorroded specimen of C25 grade reached 11.74 MPa, while the minimum limit bonding strength of the corroded specimen was 6.69 MPa, and the span was 5.05 MPa. The ultimate bonding strength span of the C35 grade specimens was 6.89 MPa, while the span of the C45 grade specimens was 8.73 MPa, which indicates that the higher the concrete grade is, the greater the absolute value of the decline in the ultimate bonding strength.

**Figure 9:**
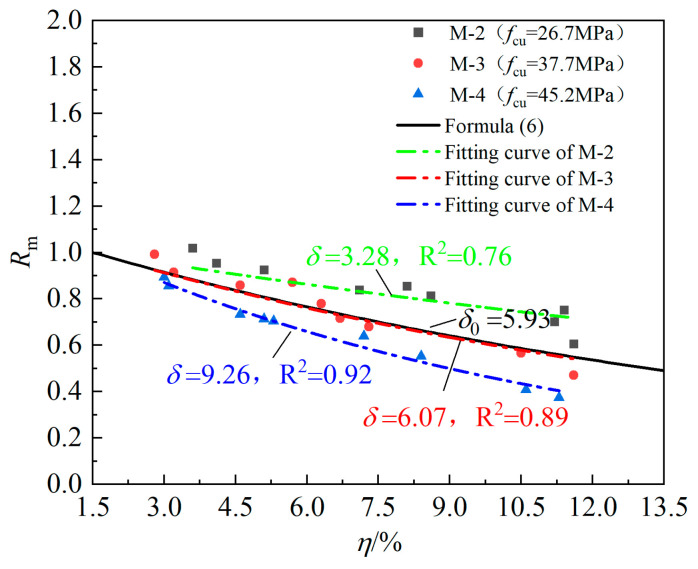
Degradation of $R_{m}$ with corrosion loss.

**Figure 10:**
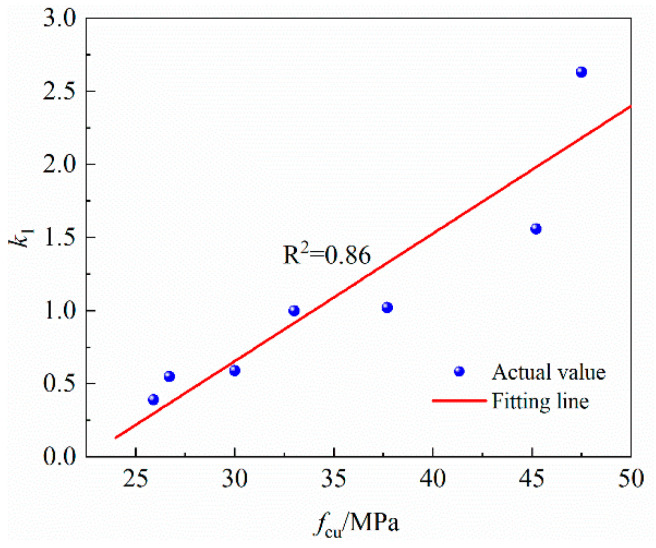
Relationship between $k_{1}$ and $f_{cu}$.

Lin et al. [16] proposed a formula for calculating the relative bonding strength by considering various factors based on the test results of 18 beam members and existing databases. As shown in Equation (6), when the corrosion loss of the longitudinal bar is less than 1.5%, the influence of corrosion on bonding strength can be negligible. The relative bonding strength deteriorates exponentially with corrosion loss. The prediction curve of the formula, the three sets of M-series data and their fitting curves are drawn in Figure 9, from which it can be found that in specimens with similar corrosion loss, the greater the concrete strength is, the lower its relative bonding strength, indicating that the higher the concrete strength grade is, the more serious the bonding strength degradation caused by the steel bar rusting. In addition, the three data fitting curves have good correlation, and the exponential form is reasonable to describe the degradation of the relative bonding strength. However, since the influence of concrete strength is not considered in Formula (7), the degradation coefficient calculated with this formula is not much different from the degradation coefficient fitted based on the M-3 series test results and there is a large deviation from the fitting values of the M-2 series and M-4 series with a maximum error of 81%, which indicates that the relative bonding strength of the specimens is also related to the concrete strength. Therefore, this paper adds a concrete strength correction coefficient $k_{1}$ on the basis of the original degradation coefficient $\delta_{0}$ in Equation (6), namely, $\delta =k_{1}\delta_{0}$. Table 3 lists the concrete correction coefficient calculated based on the three strength series here and the data of other tests presented in the reference. The concrete strength used in Formula (6) is 33 MPa, so it can be considered that the concrete correction coefficient under this strength is 1. Furthermore, it can be seen from Figure 10 that the concrete correction coefficient $k_{1}$ presents a good linear correlation with the concrete strength, and the relation is $k_{1}=0.083f_{cu}-1.82$. See Equation (8) for the revised δ formula:

(6) $$\begin{array}{c} R_{m}=\left\{\begin{array}{c} \;1\;\eta \leq \;1.5\%\;\\ e^{-\delta_{0}\left(\eta -1.5\%\right)}\;\eta >1.5\% \end{array}\right.\; \end{array}$$

(7) $$\begin{array}{c} \delta_{0}=\left\{\begin{array}{c} \;\frac{13.28-0.57\left(c/d\right)}{43.54\xi_{st}+1}\;i_{corr}\leq 200\;\mu A/{cm}^{2}\\ \frac{13.28-0.57\left(c/d\right)}{43.54\xi_{st}+1}\left(0.17ln\left(\frac{i_{corr}}{200}\right)+1\right)\;i_{corr}>200\;\mu A/{cm}^{2} \end{array}\right.\; \end{array}$$

(8) $$\begin{array}{c} \delta =\left\{\begin{array}{c} \;\frac{13.28-0.57\left(c/d\right)(0.083f_{cu}-1.82)\;}{43.54\xi_{st}+1}\;i_{corr}\leq 200\;\mu A/{cm}^{2}\\ \frac{13.28-0.57\left(c/d\right)}{43.54\xi_{st}+1}\left(0.17ln\left(\frac{i_{corr}}{200}\right)+1\right)(0.083f_{cu}-1.82)\;i_{corr}>200\;\mu A/{cm}^{2} \end{array}\right.\; \end{array}$$

where $R_{m}$ is the relative bonding strength of the specimen considering only the corrosion of the longitudinal bar, equal to the ratio of the ultimate bonding strength $\tau_{u}(\eta )$ of the corroded specimen to the bonding strength $\tau_{u}(0)$ of the uncorroded specimen. $\delta_{0}$ is the degradation coefficient, which is related to the relative concrete cover c/d, stirrup coefficient $\xi_{st}$ and corrosion current density $i_{corr}$. The stirrup coefficient $\xi_{st}=A_{st}/nDS_{st}$, where $A_{st}$ is the cross-sectional area of the stirrup, n is the number of anchorage reinforcement bars, D is the diameter of the longitudinal reinforcement and $S_{st}$ is the distance between the stirrups.

### 3.3. The Influence of Stirrup Corrosion on the Ultimate Bonding Strength

**Figure 11:**
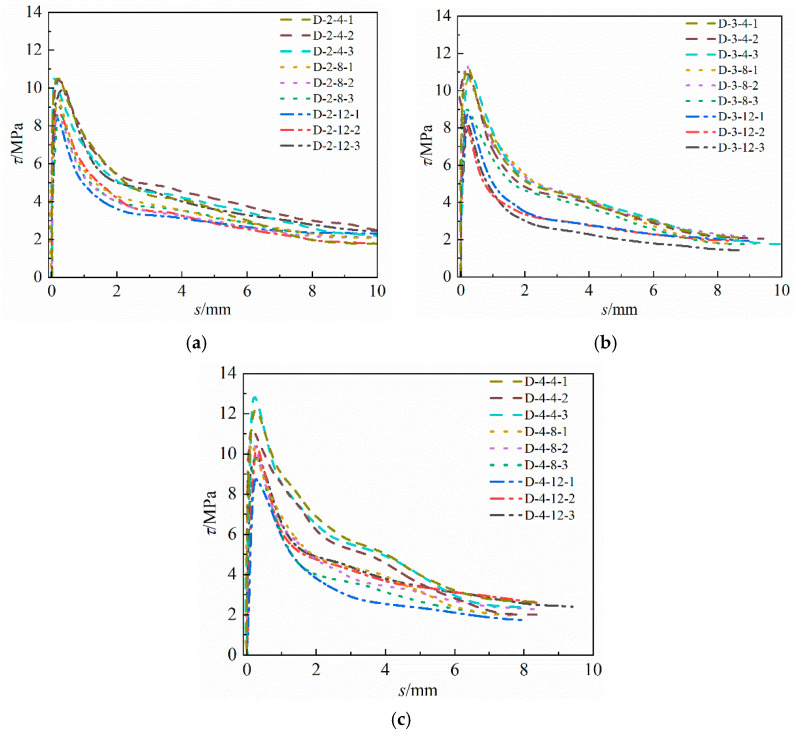
Bond–slip curves of D-series specimens. (**a**) D-2 (**b**) D-3 (**c**) D-4.

A formula for calculating the relative bonding strength considering the corrosion of longitudinal reinforcement is established above. However, stirrup corrosion is widespread in building structures in natural environments, so it is necessary to further study its influence. The results of the D-series pull-out tests are summarized in Figure 11 and Table 4. The corrosion of the inner stirrup of the specimen is more serious than that of the longitudinal reinforcement. This is because the protective layer of the stirrup is thinner and the resistivity is lower than those of the longitudinal reinforcement during the electrification process, resulting in more current flowing to the stirrup. The bond–slip curve of the D-series specimens presents a three-stage form similar to that of the M series. However, under similar longitudinal reinforcement corrosion loss, the restraint of the stirrup on the concrete is weakened due to corrosion, which further weakens the “gripping” effect of concrete on the reinforcement. Therefore, the bonding strength of the D-series specimens is lower. To obtain the quantitative relationship of the influence of stirrup rust on the bonding strength, the formula for calculating the bonding strength of both the main bar- and stirrup-corroded specimens can be set as follows:

(9) $$\begin{array}{c} \tau_{u}\left(\eta ,\eta_{st}\right)=\tau_{u}\left(0\right)R_{m}R_{st}\; \end{array}$$

where $\tau_{u}\left(\eta_{m},\eta_{st}\right)$ is the bonding strength of the specimen with the longitudinal reinforcement corrosion loss η and the stirrup corrosion loss $\eta_{st}$. The influence of the reinforcement section loss and the damage to the concrete caused by stirrup corrosion is expressed by the stirrup influence coefficient $R_{st}$.

**Figure 12:**
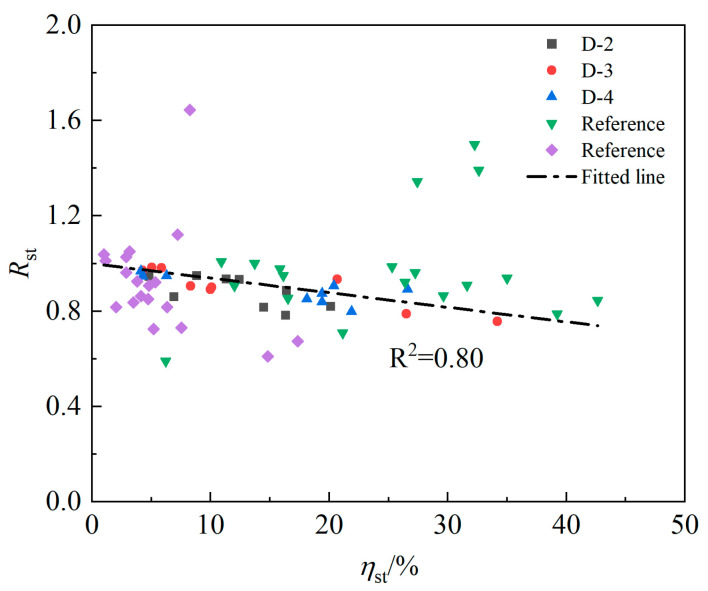
Variation in $R_{st}$ with the stirrup rusting rate. The inverted triangles are the data points from reference [12] and the diamonds are the data points from reference [20].

Based on the data of Zheng et al. [20] and Lin et al. [12] and the test results of the D-2, D-3 and D-4 series in this paper, the corresponding reduction coefficients under different stirrup corrosion loss were calculated with Equation (9), and the results are plotted in Figure 12. As shown in the figure, the effect of stirrup corrosion on the deterioration of the ultimate bonding strength cannot be ignored and can reach 25% in severe cases. Except for some test points that deviate from the fitting line due to the test accident, most test points plot around the fitting line, and the fitting line relation is $R_{st}=1-0.61\eta_{st}$. Notably, the slope of $R_{st}$ in this paper is 0.61, which is smaller than 0.68 [12] because the data used to fit the $R_{st}$ relation in Lin et al. [12] are insufficient and the test results of different concrete strengths are lacking.

### 3.4. An Improved Formula for Calculating Relative Bonding Strength

The revised relative bonding strength $R_{1}\left(\eta ,\eta_{st}\right)$ formula proposed by introducing $R_{m}$ and $R_{st}$ is shown in Equations (10)–(12). Based on the test results of 25 pull-out specimens, the empirical formula $R_{2}\left(\eta ,\eta_{st}\right)$ for relative bonding strength considering the coupling effect of longitudinal reinforcement, stirrup corrosion and concrete cover was established by Zheng et al. [20], as shown in Equation (13):

(10) $$\begin{array}{c} R_{1}\left(\eta ,\eta_{st}\right)=R_{m}R_{st}\; \end{array}\;$$

(11) $$\begin{array}{c} R_{m}=\left\{\begin{array}{c} \;1\;\eta \leq \;1.5\%\;\\ e^{-\delta \left(\eta -1.5\%\right)}\;\eta >1.5\% \end{array}\right.\; \end{array}$$

(12) $$\begin{array}{c} R_{st}=1-0.61\eta_{st}\; \end{array}$$

(13) $$\begin{array}{c} R_{2}\left(\eta ,\eta_{st}\right)=\frac{F\left(\eta ,\eta_{st}\right)\left[1+3.1e^{-0.47\left(K_{co}+33K_{st}\right)}\right]}{1+3.1e^{-0.47\left(\frac{1-\eta }{1-20.1\eta^{2}+3.247\eta }K_{co}+33\frac{1-\eta_{st}}{1+0.911{\eta_{st}}^{2}-2.266\eta_{st}}K_{st}\right)}}\; \end{array}$$

where $R_{1}\left(\eta ,\eta_{st}\right)$ and $R_{2}\left(\eta ,\eta_{st}\right)$ are the relative bonding strengths of the corrosion specimen and $F\left(\eta ,\eta_{st}\right)$ is the strength reduction coefficient. $K_{co}$ is the constraint coefficient of the concrete cover and $K_{st}$ is the constraint coefficient of the stirrup. The calculation formula of each parameter in Equation (13) is described in reference [20].

**Figure 13:**
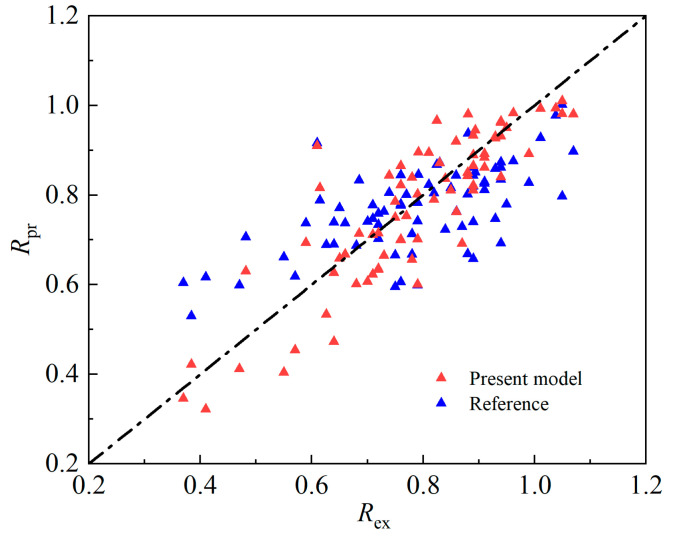
Comparison between model values and test values. The blue triangles are the data points from reference [20].

Formulas (10) and (13) were used to calculate the relative bonding strengths of the corroded specimens described in this paper and in reference [20] in Figure 13. In the figure, the abscissa $R_{ex}$ indicates the test value, and the ordinate $R_{pr}$ indicates the predicted value of the model. When the relative bonding strength is greater than 0.6, the predicted value of Equation (10) fits more closely with the actual value; when the relative bonding strength is less than 0.6, the calculated results of the model in this paper are more conservative. To compare the advantages and disadvantages of the two models more clearly, the error index *IAE* is used to quantify the accuracy of the two models. The smaller the *IAE* is, the better the predicted value of the model fits the actual value. The *IAE* can be obtained with Equation (14). The calculated *IAE* of the model in this paper is 0.08, and that of Equation (13) is 0.12. This is because the database on which this model is based not only includes references [12,20], but also includes the results of 63 specimens in this experiment. The relative bond strength calculation formula $R_{1}\left(\eta ,\eta_{st}\right)$ in this paper is more accurate.

(14) $$\begin{array}{c} IAE=\sum \frac{\left|R_{pr}-R_{ex}\right|}{\sum R_{ex}}\; \end{array}$$

Here, *IAE* is the error index, $R_{pr}$ is the predicted value of the model, and $R_{ex}$ is the test value.

### 3.5. The Improved Bond–Slip Constitutive Model

The bond–slip curve of the specimen obviously shows a rising section, falling section and a section in which the curve relatively slowly approaches a residual stress. In this paper, the piecewise constitutive model is used to describe the bond–slip behaviour of the specimen. In the constitutive model previously proposed in reference [21], the ascending section is represented by a power function, the descending section is a curve containing multiple parameters, and the residual section is modified into a straight line, whose mathematical form is as follows:

(15) $$\begin{array}{c} \tau =\left\{\begin{array}{c} \;\tau_{u}{\left(\frac{s}{s_{u}}\right)}^{\alpha }\;\left(s\leq s_{u}\right)\\ \left(1-k_{f}\right)\tau_{u}{\left(\frac{s}{s_{u}}\right)}^{\beta }+\frac{\left(1-\psi \right)k_{f}\tau_{u}}{s_{u}-s_{f}}\left(s-s_{u}\right)+k_{f}\tau_{u}\;(s_{u}<s<s_{f})\\ \;\left(1-k_{f}\right)\tau_{u}{\left(\frac{s_{f}}{s_{u}}\right)}^{\beta }+\psi k_{f}\tau_{u}\;\left(s\geq s_{f}\right) \end{array}\right.\; \end{array}$$

where $\tau_{u}$ is the ultimate bonding strength, calculated with Formula (9); α is the bonding stiffness, which is 0.25 after fitting; $s_{u}$ is the peak slip; $s_{f}$ is the residual slip; and $s_{f}$ is the distance between the ribs, which is 8 mm, as measured in this paper. β, $k_{f}$ and ψ are the parameters in the constitutive model, $\beta =-0.7{\left(c/d\right)}^{-0.24}$.

**Figure 14:**
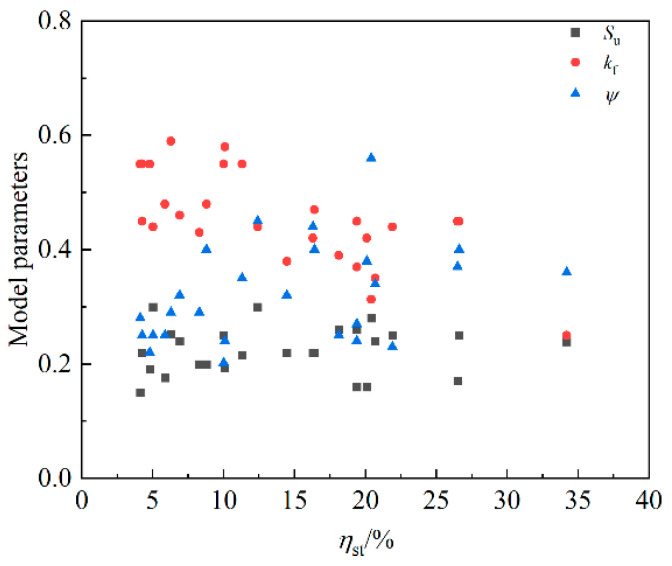
Parameters of D-series specimens.

**Figure 15:**
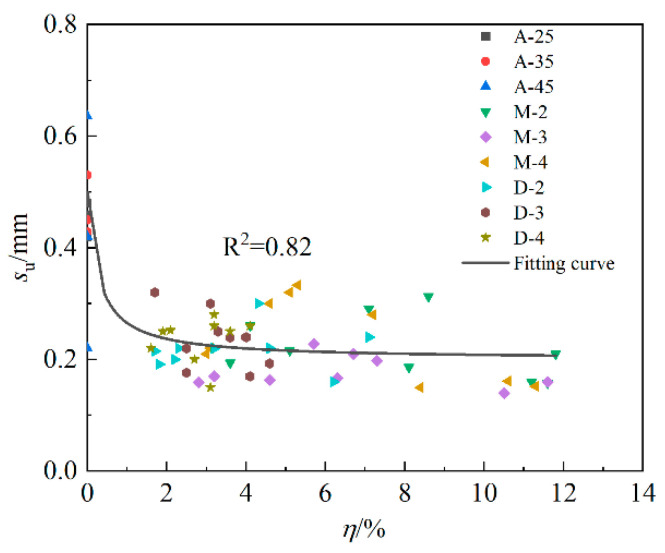
Variation in $s_{u}$ with the corrosion loss of the longitudinal bar.

Reference [21] points out that $s_{u}$ tends to decrease with the corrosion loss of longitudinal reinforcement and considers that $k_{f}$ and ψ are not affected by the corrosion of longitudinal reinforcement and can be taken as the average values. To explore the influence of stirrup rust on $s_{u}$, $k_{f}$ and ψ, the three parameters are plotted in Figure 14 along with the change in the stirrup rust rate. The distribution of $s_{u}$ did not change significantly with increasing stirrup corrosion loss, $k_{f}$ showed a slightly decreasing trend, and ψ showed a first increasing trend and then a decreasing trend. It is difficult to accurately evaluate $k_{f}$ and ψ, so $k_{f}$ and ψ of series A, M and D are approximated to the average values of the parameters of each sample in the series, as shown in Table 5. Therefore, it will produce a conservative prediction when the stirrup rust rate is high (greater than 20%). As shown in Figure 15, the calculation Formula (16) of $s_{u}$ can be obtained by fitting, and the R-square is 0.82.

(16) $$\begin{array}{c} s_{u}=\frac{71.8\eta +0.5}{358.4\eta +1}\; \end{array}$$

**Figure 16:**
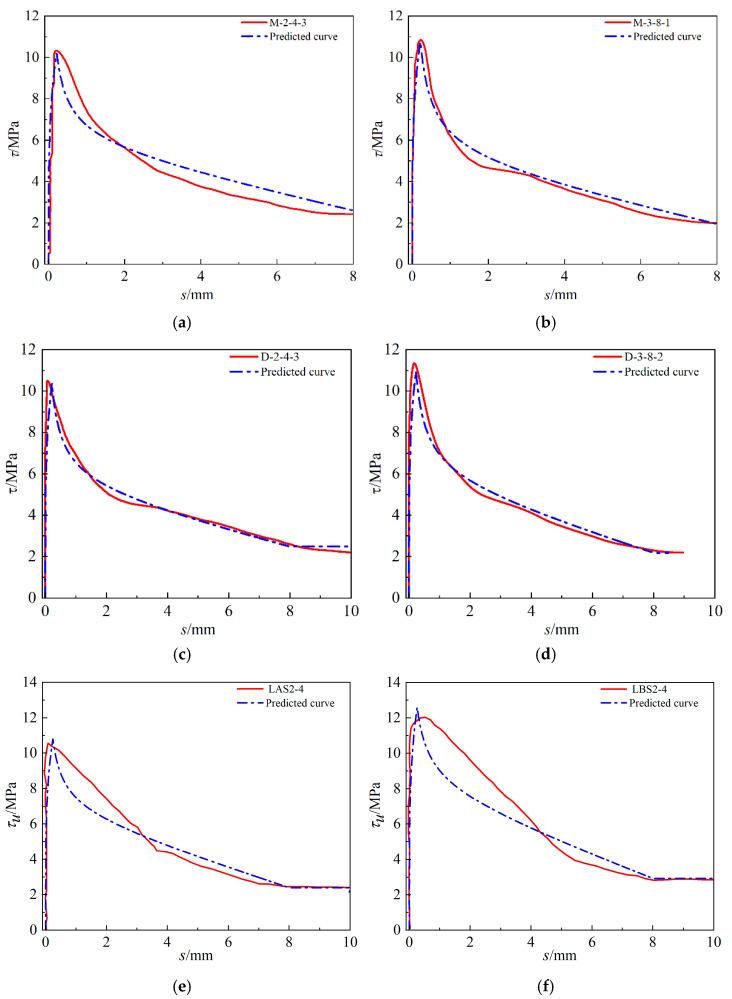
Validation of the constitutive model. (**a**) M-2-4-3, (**b**) M-3-8-1, (**c**) D-2-4-3, (**d**) D-3-8-2, (**e**) LAS2-4 and (**f**) LBS2-4. The red lines in (**e**,**f**) are the results from reference [12].

To verify the rationality of the constitutive model, several groups of corroded specimens were selected, and the test bond–slip curves and predicted curves are shown in Figure 16. The curves calculated by the constitutive model in this paper are in good agreement with the actual test.

## 4. Conclusions

Three reinforced concrete specimens with concrete strengths of 26.7 MPa, 37.7 MPa and 45.2 MPa were tested, and the degradation of the bond–slip behaviour of corroded reinforced concrete was studied by conducting electrochemically accelerated corrosion tests and pull-out tests. The following conclusions were obtained:

(1) With increasing corrosion time, different degrees of pitting and regional corrosion of the longitudinal bars are observed. The corrosion of the inner stirrup of a specimen with both longitudinal bar and stirrup corrosion is generally more serious than that of the longitudinal reinforcement, and the distribution of surface cracks is more complex than that of a specimen with only longitudinal reinforcement corrosion, showing a pattern of interlacing cracks, and the phenomenon of rusting during the corrosion process is more serious.
(2) Steel bar corrosion in a specimen will reduce the bonding strength of the specimen. The higher the concrete strength grade of the specimen is, the faster the bonding strength deteriorates with increasing corrosion loss. In addition, under the same longitudinal reinforcement corrosion loss, the bonding strength of the longitudinal reinforcement- and stirrup-corroded specimens is significantly lower than that of the longitudinal reinforcement-corroded specimens only. In severe cases, the bonding strength of the longitudinal reinforcement-corroded specimens is only 75% of that of the longitudinal reinforcement- and stirrup-corroded specimens. Moreover, the reduction effect caused by stirrup corrosion can change approximately linearly with stirrup corrosion loss.
(3) Based on the experimental results and previous work, a relative bonding strength degradation model and a bond–slip constitutive model of corroded reinforced concrete are established, accounting for many factors, such as concrete strength and longitudinal bar and stirrup rust. The empirical formula of peak slip and the values of each parameter are given, and the constitutive model is in good agreement with the relevant experimental results.
(4) The model proposed in this paper is only applicable to the case of steel bars that do not yield. In future research, the bond–slip constitutive model of corroded reinforced concrete under the yield state of steel bars could be explored.

## Figures and Tables

**Table 1:** Parameters of the specimens.

Specimen Name	Stirrup Insulation	Concrete Strength	Expected Corrosion Loss	Number of Specimens
A-25	Y	C25	0%	3
M-2-4/8/12	Y	C25	4%/8%/12%	3/3/3
D-2-4/8/12	N	C25	4%/8%/12%	3/3/3
A-35	Y	C35	0%	3
M-3-4/8/12	Y	C35	4%/8%/12%	3/3/3
D-3-4/8/12	N	C35	4%/8%/12%	3/3/3
A-45	Y	C45	0%	3
M-4-4/8/12	Y	C45	4%/8%/12%	3/3/3
D-4-4/8/12	N	C45	4%/8%/12%	3/3/3

‘Y’ means that no current passes through the stirrup, and ‘N’ means that current passes through the stirrup. A-XX indicates the uncorroded control group, where XX represents the concrete grade. M-X-4/8/12 indicates only longitudinal reinforcement corrosion, where X represents the concrete strength, 2 represents C25, 3 represents C35, 4 represents C45 and 4/8/12 corresponds to different design corrosion loss. The D series represents longitudinal bar and stirrup corrosion, where the other symbols are the same as those for the M series.

**Table 2:** Test results of the A-series and M-series specimens.

Specimen Name	Expected Corrosion Loss/%	Actual Corrosion Loss/%	Ultimate Bonding Strength/MPa	Relative Bonding Strength
A-25-1	0.00	0.00	10.80	1.00
A-25-2	0.00	0.00	11.74	1.00
A-25-3	0.00	0.00	10.92	1.00
M-2-4-1	4.00	4.10	10.63	0.95
M-2-4-2	4.00	3.60	11.35	1.01
M-2-4-3	4.00	5.10	10.32	0.93
M-2-8-1	8.00	7.10	9.37	0.84
M-2-8-2	8.00	8.60	9.03	0.81
M-2-8-3	8.00	8.10	9.48	0.85
M-2-12-1	12.00	11.20	7.80	0.70
M-2-12-2	12.00	11.40	8.36	0.75
M-2-12-3	12.00	11.60	6.69	0.60
A-35-1	0.00	0.00	12.32	1.00
A-35-2	0.00	0.00	12.74	1.00
A-35-3	0.00	0.00	12.17	1.00
M-3-4-1	4.00	2.80	12.31	0.99
M-3-4-2	4.00	3.20	11.34	0.91
M-3-4-3	4.00	4.60	10.65	0.86
M-3-8-1	8.00	5.70	10.80	0.87
M-3-8-2	8.00	6.30	9.67	0.78
M-3-8-3	8.00	6.70	8.88	0.72
M-3-12-1	12.00	7.30	8.44	0.68
M-3-12-2	12.00	10.50	7.02	0.57
M-3-12-3	12.00	11.60	5.85	0.47
A-45-1	0.00	0.00	13.83	1.00
A-45-2	0.00	0.00	13.68	1.00
A-45-3	0.00	0.00	13.53	1.00
M-4-4-1	4.00	3.00	12.22	0.89
M-4-4-2	4.00	3.10	11.70	0.85
M-4-4-3	4.00	4.60	11.39	0.73
M-4-8-1	8.00	5.30	9.63	0.70
M-4-8-2	8.00	5.10	10.85	0.71
M-4-8-3	8.00	7.20	8.73	0.64
M-4-12-1	12.00	8.40	7.54	0.55
M-4-12-2	12.00	10.60	5.97	0.41
M-4-12-3	12.00	11.30	5.10	0.37

**Table 3:** Concrete correction coefficient $k_{1}$.

Data Source	Concrete Strength/MPa	$\mathrm{Predicted}\;\mathrm{Value}\;\mathrm{of}\;\delta_{0}$	Actual Value of δ	$k_{1}$
Ref. [17]	25.9	12.60	4.87	0.39
Ref. [18]	30.0	14.49	8.61	0.59
Ref. [19]	47.5	4.99	12.98	2.60
M-2	26.7	5.93	3.28	0.55
M-3	37.7	5.93	6.07	1.02
M-4	45.2	5.93	9.26	1.56

**Table 4:** Test results of D-series specimens.

Specimen Name	Longitudinal Bar Corrosion Loss/%	Stirrup Corrosion Loss/%	Ultimate Bonding Strength/MPa	Relative Bonding Strength
D-2-4-1	1.80	4.78	10.50	0.94
D-2-4-2	1.70	11.30	10.40	0.93
D-2-4-3	2.20	8.80	10.44	0.94
D-2-8-1	4.60	16.40	9.33	0.84
D-2-8-2	3.20	16.30	8.47	0.76
D-2-8-3	2.30	14.47	8.98	0.81
D-2-12-1	6.20	20.10	8.37	0.75
D-2-12-2	7.10	6.90	8.63	0.78
D-2-12-3	4.30	12.40	9.87	0.89
D-3-4-1	2.50	4.27	11.24	0.91
D-3-4-2	1.70	8.30	11.08	0.89
D-3-4-3	3.10	5.03	10.90	0.88
D-3-8-1	3.30	10.00	9.76	0.79
D-3-8-2	2.50	5.86	11.35	0.91
D-3-8-3	4.60	10.08	8.98	0.72
D-3-12-1	4.00	20.70	8.74	0.79
D-3-12-2	4.10	26.50	8.16	0.66
D-3-12-3	3.60	34.20	8.11	0.65
D-4-4-1	2.10	6.28	12.18	0.89
D-4-4-2	3.10	4.12	11.21	0.82
D-4-4-3	1.60	4.27	12.81	0.94
D-4-8-1	1.90	21.90	10.46	0.76
D-4-8-2	2.70	19.40	10.54	0.77
D-4-8-3	3.20	18.12	9.75	0.71
D-4-12-1	4.10	19.40	8.74	0.64
D-4-12-2	3.20	20.40	10.37	0.76
D-4-12-3	3.60	26.60	9.79	0.72

**Table 5:** Values of parameters $k_{f}$ and ψ.

Series	$k_{f}$	ψ
A	0.45	0.27
M	0.51	0.41
D	0.46	0.32

## Data Availability

Data are contained within the article.
